# Stereotactic radio-neurosurgery for jugular foramen schwannomas

**DOI:** 10.1007/s00701-024-06211-x

**Published:** 2024-08-23

**Authors:** Camil Bourhila, Cristian Cotrutz, Roy Thomas Daniel, Mercy George, Luis Schiappacasse, David Patin, Marc Levivier, Constantin Tuleasca

**Affiliations:** 1 Neurosurgery Service and Gamma Knife Center, Lausanne University Hospital (CHUV), Rue du Bugnon 44-46, BH-08, CH-1011 Lausanne, Switzerland; 2 Faculty of Biology and Medicine (FBM), University of Lausanne (UNIL), Lausanne, Switzerland; 3 Ecole Polytechnique Fédérale de Lausanne (EPFL, LTS-5), Lausanne, Switzerland; 4 ENT Department, Lausanne University Hospital (CHUV), Lausanne, Switzerland; 5 Radiation Oncology Department, Lausanne University Hospital (CHUV), Lausanne, Switzerland; 6 Institute of Radiation Physics, Lausanne, Switzerland

**Keywords:** Jugular foramen, Schwannoma, Cranial nerves, Stereotactic radiosurgery, Gamma Knife

## Abstract

**Background:**

Stereotactic radiosurgery (SRS) represents a minimally invasive and valuable alternative for jugular foramen schwannomas (JFS), both as upfront and/or adjuvant treatment (in hybrid approaches).

**Methods:**

We conducted a retrospective review of our cases treated at the Lausanne University Hospital (CHUV) from June 2010 to October 2023. Eleven patients underwent SRS, among whom three had prior surgery, two in our center in the frame of a planned combined approach and one in another center. Two patients received "volume-staged" SRS. The mean age at SRS was 60 years (median 68; range 29–83). Cranial nerve (CN) symptoms were present in six patients, while five were asymptomatic. The mean tumor volume at SRS was 2.1 cc (median 1.2; range 0.068–7.3 cc), with a 12 Gy marginal dose prescribed in all cases.

**Results:**

The mean follow-up period was 3.9 years (median 2, range 1–7). Cranial nerve function improved after SRS in six patients, while five remained stable. At the last follow-up, all tumors showed a decrease in volume, except for one patient, who underwent surgery at 18 months after SRS, for volumetric increase at 6 and 12 months, with further XII^−th^ CN palsy and medulla oblongata compression. Although tumor decreased at 18 months, such patient needed microsurgical resection for symptom persistence and was further controlled. The mean tumor volume at 1 year post-SRS was 1.6 cc (median 0.55; range 0.028–7.77 cc), at 2 years was 1.31 cc (median 0.76; range 0.19–5), and at 3 years was 1.32 cc (median 0.59; range 0.23–4.8). No adverse radiation events were observed.

**Conclusions:**

Stereotactic radiosurgery is considered a safe and effective treatment for jugular foramen schwannomas, ensuring high rates of tumor control in all patients over the long term. The cranial nerve function improved after SRS in the 6 patients who had deficits and the other 5 patients who had no deficits remained asymptomatic. For larger tumors, combined/hybrid approaches can be a valuable alternative, to obtain tumor control and to preserve neurological function.

## Introduction

Jugular foramen tumors, including paragangliomas, schwannomas, and meningiomas, are rare [8]. Schwannomas of cranial nerves IX, X, and XI (glossopharyngeal, vagus, and accessory nerves) are particularly uncommon, accounting for approximately 2.9% to 4% of all intracranial schwannomas [9]. They constitute 10 to 30% of all tumors around the jugular foramen [2]. These tumors, mainly lower cranial nerve schwannomas (LCNS), pose diagnostic challenges, often requiring confirmation of the nerve of origin through intraoperative neurophysiological monitoring or direct inspection [1]. Historically, aggressive surgical resection has been the standard treatment. However, this anatomical area is particularly challenging due to the complex anatomy and the involvement of various skull-base structures, predominantly the lower cranial nerves (CN) [1]. Upfront surgical resection is needed for large tumors with symptomatic mass effect, brainstem compression or hydrocephalus [3]. Common postoperative effects include speech or swallowing deficits, related to glossopharyngeal and vagal injuries [3]. Recently, surgical approaches have become more conservative, due to the increasing use of the radiation techniques, including stereotactic radiosurgery (SRS) [3].

Stereotactic radiosurgery is a valuable minimally invasive alternative, which can be used either as upfront technique or in the frame of adjuvant treatments, after prior resection [4, 6, 7].

In the present study, we retrospectively review our experience of SRS for jugular foramen schwannomas (JFS), treated with SRS as initial treatment or combined with surgery, in the frame of hybrid approaches. We focus on tumor control and clinical outcomes.

## Material and methods

### Study design

The study was unicentric, retrospective, non-randomized. Eleven patients were treated at the Lausanne University Hospital, Switzerland, between May 2013 until October 2023.

The Ethical Committee of the Lausanne University Hospital and University of Lausanne approved the study (CER-VD 2023–01706).

### Patient population

Basic demographic data can be found in Table 1.

**Table 1:** Basic demographic and clinical outcomes

Case No	Age	Sex	Upfront SRS/ Combined/ Staged	Preexisting Deficits	Gardner-Robertson Pre GK	Gardner-Robertson Post GK	Deficits at Last Follow up
1	34	F	SRS	Disabling diplopia. paresis of CN III on the right, right hypopharingeal salivary stasis. moderate dysphonia. dysphagia	1	1	Paresis of CN III on the right and dysphonia: stable Right hypopharingeal salivary stasis / dysphagia: Improvement
2	83	M	SRS	Swallowing disorder, damage to the CN XI with muscle damage to the SCM and trapezius	1	1	Disappearance of swallowing disorder 12 months after GK
3	72	M	SRS	Dysgeusia, glossodynia and mouth burns	1	1	Asymptomatic (Disappearance of symptoms 10 months after GK)
4	60	F	SRS	Asymptomatic	1	1	Asymptomatic
5	39	F	Staged SRS	Right hemi laryngeal with neurogenic damage probably related to the right X NC, dysphonia, dysphagia, hoarse voice	1	1	Asymptomatic (Disappearance of symptoms 12 months after GK)
6	69	F	SRS	Asymptomatic	1	1	Asymptomatic
7	53	F	Surgery at another institution + Staged SRS + Surgery	Difficulty chewing but not for swallowing, damage to the right XII NC with progressive derivation of its right hemi-tongue, right base vagal paresis	1	1	Tongue deviation to the right: Improvement Pseudoswelling at 6 months, which continued at 1 year with further volumetric increase and XII^th^ CN palsy
8	29	M	Combined: Surgery + SRS	Asymptomatic	1	1	Asymptomatic
9	71	F	Combined: Surgery + SRS	Asymptomatic	1	1	Asymptomatic
10	68	F	SRS	Balance disorders, tongue deviation to the left, right hyporeflexia	1	1	Balance disorders: Improvement Tongue deviation to the left: stable
11	83	F	SRS	Asymptomatic	1	1	Asymptomatic

There were eight women and three men. The mean age at the time of SRS was 60 years (range, 29–83). The mean follow-up period was 3.9 years (median 2, range 1–7).

Cranial nerve (CN) symptoms (dysphonia / glossodynia / dysgeusia / dysphagia / hemilaryngeal paralysis) were present in six patients, while five were asymptomatic.

### Patient diagnosis and selection for treatment

Pretherapeutic brain MRI was performed in all cases. It was additionally discussed with our neuroradiology team, which confirmed the diagnosis of JFS. Furthermore, all cases were considered in the frame of a multidisciplinary team (neurosurgery, ENT and speech therapist). Two cases benefited from a planned combined procedure, in the frame of a hybrid approach, with initial scheduled subtotal microsurgical resection at our institution followed by SRS on the remanent tumor.

### Gamma knife radiosurgery procedure

After placement of the Leksell stereotactic model G frame (Elekta Instruments, AB, Sweden) under local anesthesia, all patients underwent MRI with the following sequences: a T1-weighted native and gadolinium-enhanced and a T2-weighted axial CISS/Fiesta 3D (for better cranial nerve visualization and T1 Vibe (0.5 mm thickness). Bone-windowed computer tomography supplemented the MRI in all cases. Radiosurgery was performed using the Leksell Gamma Knife Perfexion (2010–2016) and following, the ICON (2016–2023, Elekta Instruments, AB, Sweden).

Dosimetric data can be found in Table 2. The mean tumor volume (TV) was 2.1 cc (median 1.2; range 0.068–7.3 cc). The marginal dose was 12 Gy in all cases, at a prescription isodose of 50% in eight cases, 60% in one case, 55% in one other case and 52% in one other case. The mean isodose was 51% (median 50%, range 50–60%).

**Table 2:** Dosimetric data for each patient

Case No.	Target volume (TV) (cc)	Marginal Dose (Gy) / Prescription Isodose (%)	Number of isocenters	PIV (volume of the matrix receiving the prescription isodose)	TVPIV(Target volume covered by PIV)	PIV 0.5 (volume of the matrix receiving the half prescription isodose)	Max dose received by structures at risk (Gy) (Left (L). Right (R))	Coverage	Conformity	Selectivity	Index Gradient
1	0.649	12/50	14	0.83	0.64	2.88	Lens L & R: 0.2 Chiasma: 0.2 Brainstem: 1.9 Cochlea: 0.9	0.99	1.27	0.78	3.48
2	0.552	12/50	9	0.82	0.55	2.41	Lens L & R: 0 Chiasma: 0.1 Brainstem: 2.0 Cochlea: 2.3	1.00	1.49	0.67	2.93
3	0.659	12/50	9	0.89	0.65	2.84	Lens L & R: 0 Chiasma: 0.2 Brainstem: 4.0 Cochlea: 0.3	0.99	1.34	0.74	3.21
4	0.600	12/50	7	0.73	0.59	2.02	Lens L & R: 0 Chiasma: 0.4 Brainstem: 11.4 Cochlea: 1.3	0.99	1.21	0.82	2.79
5	Stage 1: intracranial portion: 2.120 Stage 2: extracranial portion: 1.275	12/50	Stage 1: 21 Stage 2: 7	Stage 1: 2.64 Stage 2: 1.78	Stage 1: 2.09 Stage 2: 1.26	Stage 1: 7.20 Stage 2: 5.80	Lens L & R: Stage 1: 0.2 / Stage 2: 0.5 Chiasma: Stage 1: 0.7 / Stage 2: 0.2 Brainstem: Stage 1: 12.3 / Stage 2: 1.2 Cochlea: Stage 1: 3.4 / Stage 2: 0.5	Stage 1: 0.98 Stage 2: 0.99	Stage 1: 1.25 Stage 2: 1.39	Stage 1: 0.79 Stage 2: 0.71	Stage 1: 2.73 Stage 2: 3.27
6	0.068	12/60	3	0.12	0.07	0.46	Lens L & R: 0 Chiasma: 0 Brainstem: < 5 Cochlea: 0.2	1.00	1.74	0.58	3.91
7	Stage 1: intracranial portion: 3.320 Stage 2: juxtacranial portion: 2.970	12/50	Stage 1: 15 Stage 2: 8	Stage 1: 3.88 Stage 2: 3.95	Stage 1: 3.29 Stage 2: 2.94	Stage 1: 10.20 Stage 2: 10.79	Lens L & R: Stage 1: 0.1 & 0.2 / Stage 2: 0.4 & 0.2 Chiasma: Stage 1: 0.1 / Stage 2: 0.1 Brainstem: Stage 1: 8.6 / Stage 2: 2.1 Cochlea: Stage 1: 1.3 / Stage 2: 2.2	Stage 1: 0.99 Stage 2: 0.99	Stage 1: 1.17 Stage 2: 1.33	Stage 1: 0.85 Stage 2: 0.74	Stage 1: 2.63 Stage 2: 2.73
8	7.300	12/50	19	8.96	7.13	23.91	Lens L & R: 0.2 & 0.3 Chiasma: 0.8 Brainstem: 9.3 Cochlea: 5	0.98	1.23	0.80	2.67
9	3.980	12/50	31	4.40	3.94	11.90	Lens L & R: 0.2 & 0.3 Chiasma: 1 Brainstem: 15.3 Cochlea: 3.5	0.99	1.11	0.90	2.70
10	3.740	12/55	41	4.83	3.74	12.33	Lens L & R: 0.3 & 0.1 Chiasma: 1.1 Brainstem: 11.9 Cochlea: 2.1	1.00	1.29	0.77	2.55
11	0.593	12/52	16	0.69	0.59	2.10	Lens L & R: 0 Chiasma: 0.1 Brainstem: 13.1 Cochlea: 0.7	0.99	1.17	0.85	3.03

The mean prescription isodose volume (PIV) was 2.6 cc (median 1.8 cc, range 0.118–8.96 cc).

Due to the anatomical location, special care was given to the fall-off of dose towards cochlea and brainstem. The mean dose received by the cochlea was 1.82 Gy (range 0.2–5). The mean dose received by the brainstem was 7.85 Gy (range 1.2–15.5). The mean dose received by the chiasma was 0.42 Gy (range 0.1–1.1).

### Single-session and “volume-staged” SRS

Nine patients received upfront and single-session GK SRS. Two patients were treated with “volume-staged” SRS. These two cases had tumors with invasions into different structures (intra-, extra-, and juxta-cranial), which would have created a relatively high volume for single-session SRS.

The timeframe between the two stages was 4 months for one case and 3 months for the other.

### Upfront SRS and combined approach

Upfront SRS was performed on eight patients. Two cases underwent a planned combined approach, due to a larger preoperative tumor volume. One patient had prior surgery in another institution and further SRS in our Center.

### Outcome measures during pre- and posttherapeutic stage

**Fig. 1:**
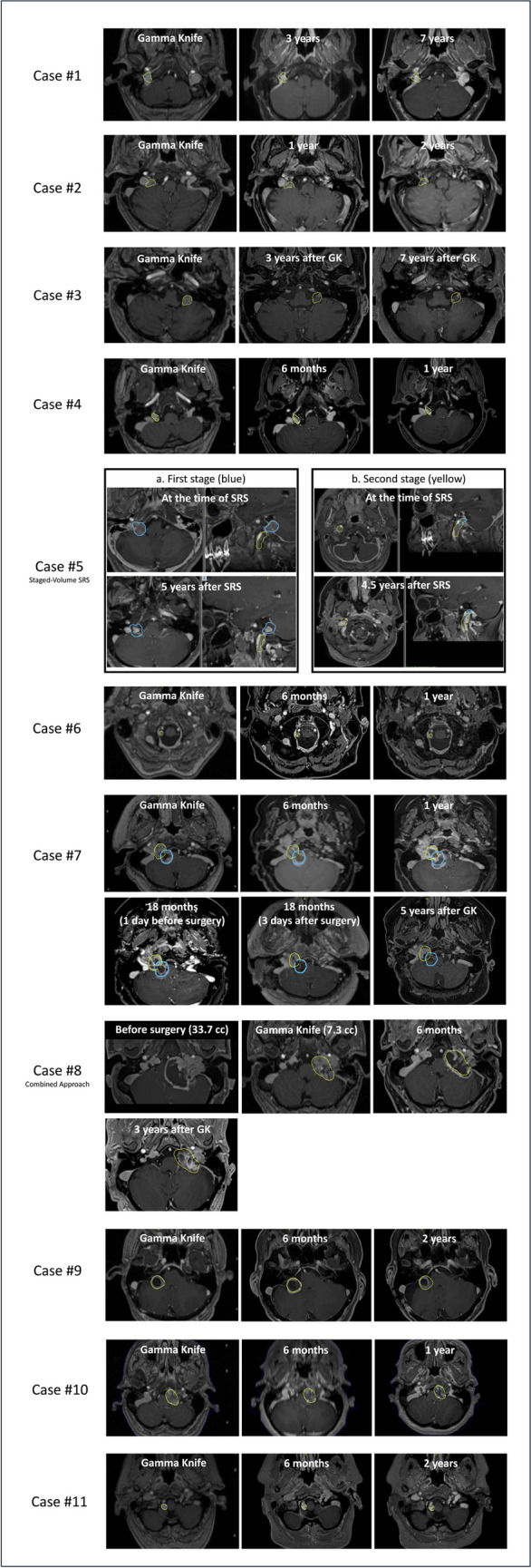
Illustration of all cases treated in Lausanne; the dosimetry is colored in yellow for single session treatments and in yellow and blue for staged volume treatments. Are depicted therapeutic images, as well as follow-up MRIs, co-registered with the therapeutic ones. For illustration purposes, we selected for detailed explanations the following: Case 3: example of an upfront SRS approach with initial tumor volume, 3 years after SRS and 7 years after SRS (with major decrease and 20.8% from the initial volume); Case 5: example of a volume-staged radiosurgery, with the first stage (**a**) and second stage (**b**). Case 8: example of a combined approach with preoperative volume, at the time of SRS, 6 months after SRS with transient tumor swelling and further decrease at 3 years after SRS

Each patient benefitted from a regular clinical and radiologic assessment, which took place at baseline (pretherapeutically) and during follow-up course at 6, 12, 24, 36, 60 and 84 months. For a detailed view of all the cases treated in Lausanne, please see Fig. 1.

Tumor volume was drawn individually, for each patient and each time-point after SRS. The follow-up MR sequence was imported within the Leksell Gamma Plan and further co-registered with the therapeutic one. The selected MR sequence was T1 Gadolinium 1-mm slice. Volume was draw by hand, using the automatic segmentation tool initially, and further refined using the manual module.

## Results

### Radiological outcome after SRS

**Fig. 2:**
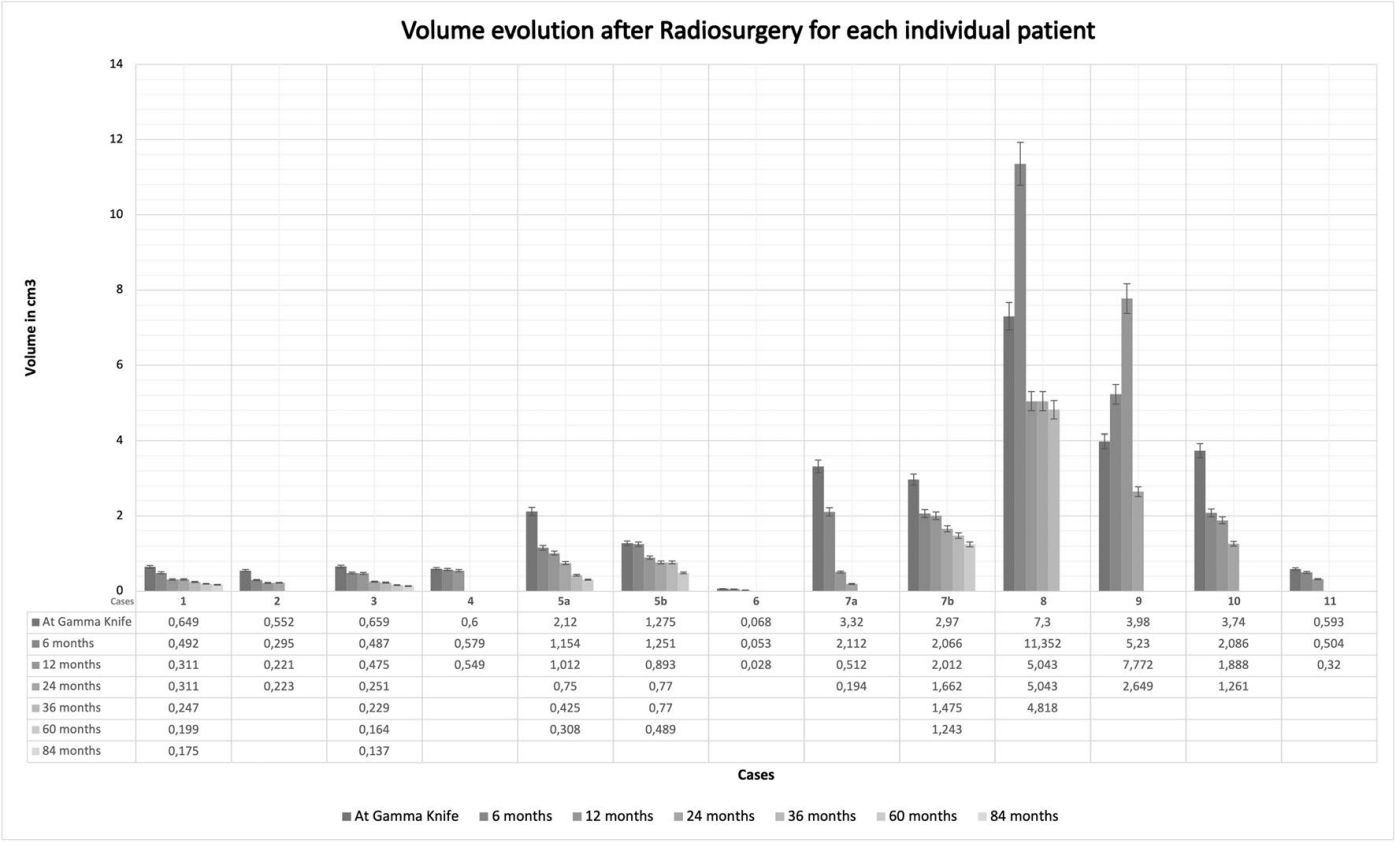
Volume evolution after radiosurgery for each individual patient. The cases are numbered from 1 to 11. The letters “a” and “b” correspond respectively to “stage 1” and “stage 2” for each patient

At the last follow-up, tumor control was achieved in 10 out of 11 cases, with all 10 being decreased in size. For a detailed view of all the tumor volumes at the respective time-points please see Fig. 2.

One patient had a minimal transient tumor increase at 6 months, which continued at 1 year after SRS for a right XII-th nerve schwannoma with intracanalicular and cervical extension, with medulla oblongata compression; due to additional cranial nerve dysfunction and such further increase at one year, despite a volumetric decrease at 18 months after SRS, she needed to undergo microsurgical resection (for details, please see Case #7 in Fig. 1).

Otherwise and during follow-up course, there was a continuous volumetric decrease starting 1 year after SRS, while the mean tumor volume at the time of SRS was 2.1 cc (median 1.2; range 0.068–7.3 cc), at 1 years after SRS was 1.6 cc (median 0.55; range 0.028–7.77 cc), at 2 years was 1.31 cc (median 0.76; range 0.19–5) and at 3 years was 1.32 cc (median 0.59; range 0.23–4.8).

### Clinical outcome after SRS

The cranial nerve function improved after SRS in the 6 patients who had deficits and the other 5 patients who had no deficits remained asymptomatic.

The delay in cranial nerve improvement ranged between 30 days for a patient with swallowing problems and up to 1 year for dysphonia.

All patients experienced an overall improvement in quality of life.

### Complications after SRS

No patient experienced adverse radiation events (ARE). However, one patient had a minimal transient tumor increase at 6 months, which continued at 1 year after SRS for a right XII-th nerve schwannoma, with medulla oblongata compression and cranial nerve dysfunction. Despite tumor volume regression at 18 months after SRS, due to symptom persistence, she underwent microsurgical resection. This was thus a transient tumor expansion.

## Discussion

Here, we present our experience with SRS for a rare pathology, JFS, over a period of 13 years and comprising 11 cases. Our data show 10 out of 11 at tumor control, with a decrease in volume at last follow-up for all 10 cases. However, one patient had a minimal transient tumor expansion at 6 months and 12 months after SRS, with further decrease at 18 months, for a right XII-th nerve schwannoma with intracanalicular and cervical extension; due to symptom persistence, the patient needed to undergo further microsurgical resection. With regards to the clinical outcome, cranial nerve function improved in 6 patients, while none experienced clinical deterioration. The delay in cranial nerve improvement ranged between 30 days for a patient with swallowing problems and up to 1 year for dysphonia. There was no ARE.

Jugular foramen schwannomas were initially referred for upfront surgical resection. In a recent series, Aftahy et al. [1] reported a gross total resection (GTR) in a series of 9 patients, via either a retrosigmoid or extreme lateral infrajugular transcondylar approach. However, 33.3% suffered from a new permanent neurological deficit, while one patient had a facial palsy, and 2 transient hoarseness [1]. Carlstrom et al. [3] analyzed 34 patients who underwent surgical resection and 24 who underwent SRS, with larger tumor size as indication for surgery. GTR was obtained in only 52%, with remanent tumor located at the level of the jugular foramen in most cases [3]. The authors found that post-treatment worsening of symptoms occurred more frequently with surgery (68%) as compared to SRS (29%) [3]. At follow-up, tumor control was 97% in the surgical cohort and 96% among SRS patients [3]. The authors concluded that adjuvant SRS can be a valuable alternative in this indication [3].

The present data confirms the available previous clinical research results reported for SRS for JFS. The largest multicentric study included 92 patients [4] treated in 9 centers, of whom 41 had prior surgical resection. After a median follow-up of 51 months, and a median marginal dose of 12.5 Gy (10–18), stability or regression was found in 80 (87%) and progression in 12 (13%) patients. Peker et al. [7] reported 17 patients, with a tumor growth control rate of 100% and 35% clinical improvement. Kim et al. [5] reported a higher incidence of ARE of 35%, after treatment of 46 patients with a marginal dose of 12–14 Gy, with predominantly cranial nerve dysfunction, not permanent and mostly improved with corticosteroid treatment. In univariate analysis, dumbbell-shaped tumors and initial tumor volume were meaningfully linked with the occurrence of AREs [5]. Zhou et al. [10] reported the results for 74 cases treated with hypofractionated stereotactic radiotherapy (HSRT) by Cyberknife for larger tumor volumes (range 0.5–41 cc, median 14.8), while prescribing 18.2 Gy/2 fractions, 21.0 Gy/3 fractions, and 21.6 Gy/4 fractions. Tumor stability or regression was found in 93.2%, while 6.8% experienced tumor progression [10]. Preexisting cranial nerve neuropathies improved in 62% of cases.

Our study has several inherent limitations. The first is its retrospective nature, with all the biases derived from that. The second is the low number of cases, precluding further detailed statistical analysis in this rare pathology.

## Conclusion

Stereotactic radiosurgery is a valuable alternative as upfront or adjuvant treatment in the frame of planned combined approaches for JFS. A marginal dose of 12 Gy, by analogy with the one used for vestibular schwannomas, ensures high tumor control rates and symptomatic improvement. For JFS with both intra- and extracranial compartment, a volume-staged SRS was successfully performed in our experience.

## Data Availability

N/A.

## Code availability

N/A.
